# Editorial: Application of Next Generation Sequencing (NGS) in Infection Prevention

**DOI:** 10.3389/fpubh.2022.945595

**Published:** 2022-06-09

**Authors:** Michael Kemp, Martin Maiers

**Affiliations:** ^1^Department of Regional Health Research, Faculty of Health Sciences, University of Southern Denmark, Odense, Denmark; ^2^Marrow Donor Program, Minneapolis, MN, United States

**Keywords:** next generation sequencing, NGS, infection prevention, disease surveillance, antibiotic resistance, infection control

In the past decade, Next Generation Sequencing (NGS) has become widely applied for diagnostics, surveillance, and research of infectious diseases. In 2017, Deurenberg et al. published a review on application of NGS in clinical microbiology and infection prevention (1). Since then, the field has moved fast. The continuous development of sequencing technology and data handling software has made NGS accessible even to smaller laboratories. NGS data are easily shared, allowing collaboration on *in silico* analyses between institutions. Both microorganisms and host responses may be analyzed by NGS. Thus, NGS offers new options for current and future prevention of infections. The aim of this Research Topic is to illustrate various uses of NGS in prevention of infections by virus, bacteria, and parasites.

The emergence of new variants during the SARS-CoV-2 pandemic has clearly demonstrated the importance of NGS based surveillance of viral diseases. However, NGS has also revealed other discoveries on the epidemiology of SARS-CoV-2. Wang et al. report co-existence of genetically distinct viruses within a host and a narrow transmission bottleneck between patients from the same households using viral whole genome sequencing (WGS). This finding is important in understanding the population dynamics of SARS-CoV-2.

For the study of bacteria, Rogers et al. designed a scoping review to determine the value of WGS in the surveillance of antibiotic resistance in enterococci. These are important bacteria, as *Enterococcus faecalis* and *E. faecium* were each responsible for between 100.000 and 250.000 deaths associated with antibiotic resistance globally in 2019 (2). Rogers et al. conclude that WGS has been used with success, especially for detection of new genes and typing of isolates of enterococci. WGS-based typing and resistance gene detection was used by Marbjerg et al. to characterize vancomycin-resistant *E. faecium* in a hospital over 5 years. Using an online software solution that does not require knowledge in bioinformatics, the authors detected accumulation of specific clusters over time. Mao et al. used pulsed field gel electrophoresis and WGS to describe the epidemiology of multidrug-resistant *Acinetobacter baumanii* in an intensive care unit (ITU). The high discriminatory power of WGS established transmission as the main mode of acquisition of multidrug-resistant *A. baumanii* by patients in the ITU. Surveillance at the local/regional level may benefit from WGS as discussed in the review by Asare et al.. The authors evaluate the relevance of genomic epidemiology in surveillance of tuberculosis in West Africa with special focus on drug resistant strains of *Mycobacterium tuberculosis*.

Although WGS shows superior performance compared to almost any other microbial typing system, replacement of previously used methods requires translation of WGS data into the previously used system, especially during a transition phase. Examples of established tools for predicting *Salmonella enterica* serotypes, *E. coli* serotypes, Fimtypes, and FumCtypes, *S. aureus* SPAtypes, and *Pseudomonas aeruginosa* serotypes from WGS data are found on http://www.genomicepidemiology.org/services/. *Brucella* sp. are traditionally typed by Multiple Locus Variable-number Tandem Repeat Analysis (MLVA), which is a laborious methology. The work of Pelerito et al. describes the successful prediction of MLVA types from WGS data, allowing comparison of present isolates to previously typed isolates of the bacterium. Qiu et al. used an established genome database to compare a patient isolate of a rare *Salmonella enterica* serovar (*S. enterica* serovar Telelkebir) to published sequences of this serovar from various parts of the world. Core genome Multi Locus Sequencing Typing and single nucleotide polymorphism analyses suggested international transmission.

Application of metagenomic NGS (mNGS) for rapid diagnostics of infectious diseases is demonstrated by a case story by Zeng et al., who detected *Klebsiella pneumoniae* DNA in culture-negative cerebrospinal fluid and blood from a patient with liver abscesses and meningitis. Also using mWGS, Michel et al. describes a case of lung infection by *Ureaplasma* spp. and *Mycoplasma hominis* in a patient after lung transplantation and establishing cure of infection. The diversity of bile bacteria in cholecystitis was analyzed in four patients by Yan et al. using a combination of culture, conventional 16S rRNA sequencing and WGS. This approach brings new knowledge on the role of bile bacteria in cholecystitis.

Application of NGS for research in parasitic infections is demonstrated by He et al., who used Single Molecule Real Time sequencing and Illumina sequencing for transcriptome analyses of different developmental stages of the mite *Otodectes cynotis*. The mite infests a variety of animals, causing allergic itching external otitis. Almost 2,700 genes that were differentially expressed by adult mites and nymph/larva were detected. The analyses allowed identification of almost 400 putative allergen genes with potential importance for pathogenesis. Furthermore, the analyses indicated regulation of KEGG pathways in the two stages. The study provides new knowledge of the biology of the parasite and the nature of the disease it causes and identifies targets for further studies.

Next Generation Sequencing provides new insights into the genetics of pathogens. The papers included in this Research Topic demonstrate applications of NGS on research in basic microbiology as well as diagnostics and surveillance of infectious diseases. Both the laboratory handling and subsequent data analyses of NGS are under constant development. As NGS becomes easier, cheaper, more rapid and more scalable, it will be more widely accessible and gain an even more important role in future infection prevention.

## Author Contributions

MK was a guest associate editor of the Research Topic and drafted the paper text. MM was a guest associate editor of the Research Topic and edited the text. All authors contributed to the article and approved the submitted version.

## Conflict of Interest

The authors declare that the research was conducted in the absence of any commercial or financial relationships that could be construed as a potential conflict of interest.

## Publisher's Note

All claims expressed in this article are solely those of the authors and do not necessarily represent those of their affiliated organizations, or those of the publisher, the editors and the reviewers. Any product that may be evaluated in this article, or claim that may be made by its manufacturer, is not guaranteed or endorsed by the publisher.
